# Socio-economic consequences of mental distress: quantifying the impact of self-reported mental distress on the days of incapacity to work and medical costs in a two-year period: a longitudinal study in Germany

**DOI:** 10.1186/s12889-021-10637-8

**Published:** 2021-03-31

**Authors:** Gerhard Müller, Manuela Bombana, Monika Heinzel-Gutenbrenner, Nikolaus Kleindienst, Martin Bohus, Lisa Lyssenko, Ruben Vonderlin

**Affiliations:** 1 Department of Health Promotion, AOK Baden-Wuerttemberg, Presselstraße 19, 70191 Stuttgart, Germany; 2 Department of General Practice and Health Services Research, Heidelberg University Hospital, Heidelberg, Germany; 3 MH Statistics Consulting, Marburg, Germany; 4 Institute for Psychiatric and Psychosomatic Psychotherapy, Central Institute of Mental Health, Medical Faculty Mannheim, Heidelberg University, Mannheim, Germany; 5 McLean Hospital, Harvard Medical School, Boston, MA USA; 6 Department of Public Health and Health Education, University of Education, Freiburg, Germany

**Keywords:** Mental distress, Incapacity to work, Longitudinal study, Prevention and health promotion, Presenteeism

## Abstract

**Background:**

Mental disorders are related to high individual suffering and significant socio-economic burdens. However, it remains unclear to what extent self-reported mental distress is related to individuals’ days of incapacity to work and their medical costs. This study aims to investigate the impact of self-reported mental distress for specific and non-specific days of incapacity to work and specific and non-specific medical costs over a two-year span.

**Method:**

Within a longitudinal research design, 2287 study participants’ mental distress was assessed using the Hospital Anxiety and Depression Scale (HADS). HADS scores were included as predictors in generalized linear models with a Tweedie distribution with log link function to predict participants’ days of incapacity to work and medical costs retrieved from their health insurance routine data during the following two-year period.

**Results:**

Current mental distress was found to be significantly related to the number of specific days absent from work and medical costs. Compared to participants classified as no cases by the HADS (2.6 days), severe case participants showed 27.3-times as many specific days of incapacity to work in the first year (72 days) and 10.3-times as many days in the second year (44 days), and resulted in 11.4-times more medical costs in the first year (2272 EUR) and 6.2-times more in the second year (1319 EUR). The relationship of mental distress to non-specific days of incapacity to work and non-specific medical costs was also significant, but mainly driven from specific absent days and specific medical costs. Our results also indicate that the prevalence of presenteeism is considerably high: 42% of individuals continued to go to work despite severe mental distress.

**Conclusions:**

Our results show that self-reported mental distress, assessed by the HADS, is highly related to the days of incapacity to work and medical costs in the two-year period. Reducing mental distress by improving preventive structures for at-risk populations and increasing access to evidence-based treatments for individuals with mental disorders might, therefore, pay for itself and could help to reduce public costs.

## Background

Mental disorders are related to high individual suffering and a significantly reduced quality of life for those affected [1, 2]. In addition, they are related to significant socio-economic burdens worldwide [3–6]. Depression and anxiety disorders are ranked third and eighth among all diseases regarding the most years lived with disability (DALY) worldwide [5, 7]. About one in five people across industrialized countries and the world is suffering from a current mental disorder (18% Germany, 17.3% the EU, 18.9% the US, and 17.6% worldwide) [8, 9]. According to current forecasts, the economic burden of mental disorders will continue to increase in the coming years [10]; accordingly, it has been assumed that direct and indirect medical costs due to mental illness will more than double between 2010 and 2030 (factor 2.4) and that the global loss of economic output (loss of working days) will amount to $16.3 USD trillion during this period (2011–2030) [10, 11]. This global loss of economic output due to mental disorders is not only caused by increased absence days, but also by an increased prevalence of presenteeism. Presenteeism, which refers to attending work while ill [12] has been estimated to produce 4-times as many costs compared to being absent from work [13].

Therefore, recognizing and reducing mental distress is of central importance to society to reduce individual suffering and socio-economic burdens [14]. For doing so, individuals with mild or moderate mental distress should be given access to prevention services. In addition, individuals with severe mental distress or even mental illness should be given rapid access to specialized treatments and professional help. However, to date, mental distress or even mental illness are often detected and treated too late or not at all; only about one in five mentally ill people seek medical treatment (18.9% as 12-month prevalence) [15]. The treatment gap for mental disorders is universally large across countries [16]. For example, in a representative survey of the French population, 46.5% of participants with any type of mental disorder reported no lifetime use of mental health treatment (ranging from 35.6% for mood disorders to 56.7% for substance use disorders) [17]. Over time, mild mental distress can turn into severe mental distress – with its consequences for the duration of incapacity to work and sickness costs [18–21]. Indeed, previous studies have shown that subclinical symptoms in the general population are predictive of later mental disorders. Subclinical psychotic symptoms, for example, were studied early in the general population [22–24], with significant associations with progression to psychotic disorders [25, 26] but also with non-psychotic disorders [27, 28]. Further studies on other subclinical symptoms suggest that ‘sadness’ as a subclinical symptom is predictive of later major depressive disorder [29, 30].

Creating a low-threshold and rapid access to professional help requires financial investment. At the same time, when considering the reduction of days of incapacity to work and productivity losses, it could save money. Results from the English Improving Access to Psychological Therapies (IAPT; e.g., [31]) service in the UK have shown that increasing access to psychological therapies would largely pay for itself by reducing other depression and anxiety-related public costs (e.g., medical costs and productivity loss) and increasing revenues (e.g., paying taxes [32];). It has been suggested that mental health services from other countries might benefit from adopting this approach [33]. However, to the best of our knowledge, a detailed analysis of the socio-economic consequences of mental distress from other European countries in the general population is still lacking. This analysis is important in estimating the potential amount of money that could be saved by effective treatments and invested in improving services. Health insurers, politicians, employers, and other health care decision-makers should, therefore, be informed about the financial consequences of increased mental distress when thinking about implementing and improving preventive and curative structures to maintain and restore mental health [34].

### Aim of the study

This study aims to examine the impact of different degrees of self-reported mental distress as expressed in terms of subclinical anxiety and depression levels on the number of specific (due to mental disorder and burnout) and non-specific (due to any diagnosis) days of incapacity to work (DIW) as well as specific and non-specific medical costs in a two-year period in Germany.

## Method

Data from a passive control cohort of a large health promotion intervention study were used, which was conducted in 2013 and 2014 in 43 locations in Southern Germany [35–37]. The study has been registered in the German Register of Clinical Trials (DRKS) and was approved by the Ethics Review Committee at the University of Heidelberg, Germany (Study Registration: DRKS00006216). For this study, data collected in a longitudinal section were analyzed. At the beginning of the study (t0), the mental distress and the sample’s socio-demographic data were collected. The DIW data were analyzed with a latency of 1 and 2 years, respectively, starting after t0.

### Sample

A total of 34,207 policyholders of a large German health insurance company were contacted, 5549 of whom declared their willingness to participate in the study. This corresponds to a response rate of 16%. The data could not be analyzed for 861 participants because (i) the questionnaire data was incomplete or DIW data was not available because of being insured with another health insurance company (*n* = 808), (ii) consent to participate in the study was withdrawn (*n* = 20), or (iii) the questionnaire was sent out twice (*n* = 23). The education variable “still in school” (*n* = 10) was excluded because the incomplete educational status could not be included in the ranking of the education factor. Of the remaining 4688 insured persons, 1329 insured persons were not included because they were not entitled to sickness benefits (e.g., pensioners, family insured persons, rehabilitates, voluntarily insured persons not entitled to sickness benefit), and 1072 because they belonged to the experimental group in the initial study. Finally, a total sample of 2287 study participants were included in the analyses. For details, see Lyssenko et al. [35].

### Assessments

#### Mental distress

Cross-sectional mental distress was assessed with the Hospital Anxiety and Depression Scale (HADS) [38]. The HADS is a self-report questionnaire measuring anxiety and depressive symptoms with good psychometric properties [39]. The questionnaire consists of seven items for each of the two subscales, assessing the frequency of occurrence of respective symptoms on a four-point Likert-Scale, ranging from 0 = ‘not at all’ to 3 = ‘very often’. Total scores can be calculated for each subscale, ranging from 0 to 21 or an overall score for both subscales ranging from 0 to 42, which can be interpreted as a global screener of mental distress [40]. Higher values in the subscales indicate more severe anxiety or depressive symptoms. Based on the values in one of the two subscales, the degree of mental distress can be differentiated as no distress (0–7), mild distress (8–10), moderate distress (11–15), and severe mental distress (≥ 16) [41]. In addition, cut-off values are provided to distinguish between inconspicuous values and values requiring therapy. For the HADS, cut-off values apply to one of the two subscales of ≥8 (values ≥11 are considered conspicuous) [42, 43]. Therefore, a need for therapy should be clarified by further procedures, even at a low level of mental distress. A meta-analytic consideration showed an averaged sensitivity of 0.82 and specificity of 0.74 when applying a cut-off point of 8 and an averaged sensitivity of 0.56 and specificity of 0.92 when applying a cut-off point of 11 across different clinical samples [44]. In our sample, the HADS showed good reliability, with a Cronbach’s α of 0.91.

#### Days of incapacity to work

The number of specific and non-specific DIW was selected from all study participants’ routine health insurance data. As specific DIW, the days of incapacity to work due to mental illness were selected classified according to the following ICD-10 domains (International Statistical Classification of Diseases and Related Health Problems), F00-F99, “mental and behavioral disorders”, and ICD 10, Z73, “problems with regard to difficulties in coping with life”, including burnout. As non-specific DIW, all days of incapacity to work due to any ICD 10 diagnosis were selected. Both specific and non-specific DIW were retrieved cumulatively during the first and second year, starting on the day after the HADS assessment.

#### Medical costs

Direct specific and non-specific medical costs were retrieved from routine health insurance data for all study participants. The direct specific health care costs of the diagnostic main group “mental and behavioral disorders” (ICD 10, F00-F99) and “problems related to difficulties in coping with life” including burnout (Z73) were determined for the cost fields of outpatient treatment, hospital (main diagnosis), and rehabilitation (admission diagnosis). The drug costs were composed of the costs for antidepressants (N06A), psycholeptics, and psychoanaleptics in combination (N06C), anxiolytics (N05B), and hypnotics and sedatives (N05C). The averaged direct specific and non-specific medical costs are available in Euro and were retrieved cumulatively during the first and second year, starting on the day after the HADS assessment.

#### Socio-demographic data

The socio-demographic characteristics of the sample age, gender, and employment status were retrieved from the routine health insurance data. The questionnaires also assessed education and marital status.

### Statistical analyses

Many individuals do not generate any medical costs and do not cause a single DIW. Therefore, health insurance costs and DIW data are usually not normally distributed, but represent a probability distribution, with a positive mass at zero (discrete distribution) and a continuous distribution above zero. This, so-called compound Poisson-gamma distribution belongs to the family of Tweedy distributions and can be modeled in generalized linear models [45–51].

Accordingly, instead of using analyses of covariance, which would be appropriate in the case of normally distributed outcome variables, a generalized linear model with a Tweedie distribution with log link function was calculated with mental distress (HADS) as an independent variable and the specific and non-specific DIW and medical costs in the first and second year after the HADS assessment as dependent variables. Since prior studies have shown an effect of socio-demographic variables on DIW [52–54], we included our sample’s socio-demographic variables as control variables in the model (age, gender, education, and relationship and employment status). By retrieving DIW and medical costs from the routine health insurance data, these data were complete for all participants over the two-year period, so there were no dropouts. The analyses were performed with SPSS 26 (Statistical Package for Social Sciences).

## Results

The sample of 2287 participants consisted of 89% women and averaged 46.1 years of age (*SD* = 10.4). Most (72.4%) of the study participants were married. The percentage of participants holding an A-Level degree was 25.7%, whereas 2.5% had no school-leaving certificate. A small group of participants (0.5%) became unemployed by losing their job during the study period. Regarding mental distress, 47.6% of study participants were classified as no case, 24.1% were classified as a mild case, 23.5% were classified as moderate cases, and 4.7% were classified as severe cases, by applying the proposed cut-off values to their HADS scores. Socio-demographics of the sample are depicted in Table 1.

**Table 1:** Socio-demographic characteristics of the sample (*N* = 2287)

	N	Percent
Gender		
Female	2029	88.7%
Male	258	11.3%
Age		
18–33 years	366	16.0%
34–49 years	906	39.6%
50–65 years	1015	44.4%
Marital status		
Married	1655	72.4%
Not married	632	27.6%
Years of school education		
No school-leaving certificate	57	2.5%
9 years	592	25.9%
10 years	1051	46.0%
13 years (A-Level)	587	25.7%
Employment Status		
Employed	2276	99.5%
Unemployed	11	0.5%
Mental distress category (HADS)		
No case	1089	47.6%
Mild case	552	24.1%
Moderate case	538	23.5%
Severe case	108	4.7%

With 78% of all specific DIW due to mental illnesses and burnout, the diagnostic groups affective disorders (41%, e.g., depression) and neurotic, stress, and somatoform disorders (37%, e.g., anxiety disorders) dominate in the sample. None of the other diagnostic groups from the “mental and behavioral disorders” or burnout showed a proportion above 9% (i.e., 8% substance-related disorders, 7% personality disorders, 3% schizophrenia spectrum disorders, 2% problems related to difficulties in coping with life incl. Burnout, 2% behavioral syndromes associated with physiological disturbances and physical factors [e.g., eating disorders], 1% mental disorders due to known physiological conditions [e.g., dementia]).

However, 79.6% of our sample had no specific DIW in the 2 years that followed the HADS testing. Based on the sample’s HADS scores, the percentage of participants who did not have any specific DIW was 89% for no cases, 82% for mild cases, 66% for moderate cases, and 42% for severe cases. Regarding non-specific DIW, 21% of our sample had no non-specific DIW in the following 2 years. Based on the sample’s HADS scores, the percentage of participants who did not have any non-specific DIW was 27% for no cases, 20% for mild cases, 13% for moderate cases, and still 8% for severe cases.

Regarding specific (non-specific) medical costs, the proportion without any costs depending on the HADS scores was 51.1% (1.0%) for no cases, 38.9% (0.4%) for mild cases, 19.9% (0.0%) for moderate cases, and 8.3% (0.9%) for severe cases.

### Impact of socio-demographic variables

#### Gender

Gender revealed no significant differences in non-specific and specific DIW in the first and second years (Tables 2 and 3). Accordingly, no differences in non-specific medical costs were obtained between male and female participants (Table 4). However, specific medical costs were significantly increased for female participants in the second year (Table 5). Compared to men, female participants showed 1.2-times as many specific medical costs (*χ*^*2*^[1] = 2.60, *p* = .107) in the first year and 1.4-times as many specific medical costs (*χ*^*2*^[1] = 8.51, *p* = .004) in the second year.

**Table 2:** Results from the generalized linear model to predict the non-specific days of incapacity to work (due to any diagnosis) by self-reported mental distress, controlled for socio-demographic characteristics of the sample

	Non-specific DIW in the first year							Non-specific DIW in the second year						
	M	95% CI		Exp(B)	95% CI		*p*-value	M	95% CI		Exp(B)	95% CI		*p*-value
Constant				6.44	4.95	8.38	< .001				6.39	4.85	8.41	< .001
Gender														
Male^a^	32.2	22.9	45.4	1.00				22.5	15.2	33.2	1.00			
Female	37.8	27.8	51.6	1.17	0.97	1.42	.093	27.2	19.0	39.1	1.21	1.00	1.47	.052
Age groups														
18–33 years^a^	28.4	20.1	40.2	1.00				21.0	14.2	31.2	1.00			
34–49 years	35.0	25.4	48.2	1.23	1.03	1.48	.026	22.8	15.7	33.0	1.08	0.89	1.31	.412
50–65 years	42.8	31.4	58.5	1.51	1.26	1.81	< .001	31.6	22.0	45.4	1.51	1.25	1.82	< .001
Marital status														
Married^a^	33.0	24.1	45.1	1.00				23.2	16.2	33.4	1.00			
Not married	37.0	26.8	51.1	1.12	0.99	1.27	.072	26.3	18.1	38.2	1.13	0.99	1.29	.065
Years of school education														
13 years (A-Level)^a^	25.1	18.1	34.9	1.00				16.5	11.3	24.2	1.00			
10 years	30.3	22.1	41.5	1.21	1.04	1.40	.016	20.7	14.3	30.1	1.23	1.07	1.47	.005
9 years	37.9	27.5	52.2	1.51	1.23	1.79	< .001	29.6	20.3	43.0	1.79	1.50	2.13	< .001
No school-leaving certificate	51.7	34.2	78.1	2.06	1.50	2.83	< .001	36.9	23.6	57.7	2.23	1.59	3.13	< .001
Employment Status														
Employed^a^	27.9	24.6	31.5	1.00				24.9	21.9	28.3	1.00			
Unemployed	43.8	24.0	79.9	1.57	0.86	2.87	.143	24.6	12.1	49.8	0.99	0.48	2.02	.973
Mental distress category (HADS)														
No case^a^	15.8	11.4	22.1	1.00				13.1	9.0	19.1	1.00			
Mild case	25.8	18.4	36.2	1.63	1.40	1.90	< .001	19.1	13.0	28.0	1.46	1.25	1.70	< .001
Moderate case	44.7	32.2	61.9	2.82	2.45	3.25	< .001	30.6	21.1	44.3	2.34	2.02	2.71	< .001
Severe case	81.4	58.0	114.3	5.14	4.11	6.41	< .001	48.9	32.7	73.0	3.73	2.95	4.72	< .001

*Note. N* = 2.287. *t0* Assessment of predictor variables. *M* Mean days of incapacity to work, *CI* 95% confidence interval, *HADS* Hospital Anxiety and Depression Scale. ^a^reference category. Method = log-link function, Tweedie-distribution of residuals

**Table 3:** Results from the generalized linear model to predict the specific days of incapacity to work (due to mental illness) by self-reported mental distress, controlled for socio-demographic characteristics of the sample

	Specific DIW in the first year							Specific DIW in the second year						
	M	95% CI		Exp(B)	95% CI		*p*-value	M	95% CI		Exp(B)	95% CI		*p*-value
Constant				0.78	0.36	1.69	.533				1.25	0.58	2.69	.567
Gender														
Male^a^	11.2	5.1	24.5	1.00				10.2	4.4	23.3	1.00			
Female	17.5	9.1	33.9	1.57	0.91	2.69	.373	15.1	7.4	30.9	1.48	0.87	2.52	.147
Age groups														
18–33 years^a^	11.1	5.0	24.5	1.00				11.3	4.9	26.1	1.00			
34–49 years	16.3	8.1	32.5	1.47	0.89	2.41	.129	11.5	5.4	24.5	1.01	0.62	1.65	.964
50–65 years	15.2	7.7	29.7	1.37	0.83	2.26	.221	14.6	7.1	30.0	1.29	0.79	2.09	.309
Marital status														
Married^a^	14.2	7.2	28.1	1.00				11.2	5.4	23.4	1.00			
Not married	13.7	6.8	27.9	0.96	0.68	1.36	.835	13.6	6.4	29.2	1.22	0.87	1.70	.258
Years of school education														
13 years (A-Level)^a^	10.8	5.2	22.6	1.00				7.3	3.3	16.1	1.00			
10 years	13.1	6.7	25.8	1.21	0.80	1.83	.360	10.2	4.8	21.7	1.41	0.92	2.15	.114
9 years	12.1	6.0	24.6	1.12	0.70	1.79	.636	13.1	6.0	28.4	1.80	1.13	2.85	.013
No school-leaving certificate	22.1	8.7	56.5	2.04	0.93	4.48	.074	24.2	9.5	61.6	3.32	1.51	7.28	.003
Employment Status														
Employed^a^	8.4	6.0	11.7	1.00				9.0	6.5	12.5	1.00			
Unemployed	23.4	6.6	82.5	2.80	0.80	9.86	.108	17.0	4.2	68.0	1.88	0.46	7.59	.377
Mental distress category (HADS)														
No case^a^	2.6	1.2	5.6	1.00				4.3	2.0	9.3	1.00			
Mild case	7.4	3.5	15.8	2.83	1.78	4.49	< .001	6.2	2.8	14.0	1.46	0.94	2.28	.095
Moderate case	27.5	13.6	55.9	10.50	7.04	15.67	< .001	20.1	9.5	42.4	4.71	3.21	6.90	< .001
Severe case	71.5	34.7	147.1	27.26	15.79	47.07	< .001	43.9	19.5	99.0	10.29	5.97	17.72	< .001

*Note. N* = 2.287. *t0* Assessment of predictor variables. *M* Mean days of incapacity to work, *CI* 95% confidence interval, *HADS* Hospital Anxiety and Depression Scale. ^a^reference category. Method = log-link function, Tweedie-distribution of residuals

**Table 4:** Results from the generalized linear model to predict the direct non-specific medical costs (EUR) by self-reported mental distress, controlled for socio-demographic characteristics of the sample

	Non-specific costs in the first year							Non-specific costs in the second year						
	M	95% CI		Exp(B)	95% CI		*p*-value	M	95% CI		Exp(B)	95% CI		*p*-value
Constant				1217.15	1016.57	1457.31	< .001				1302.23	1082.57	1566.46	< .001
Gender														
Male^a^	2799.9	2174.8	3604.7	1.00				3142.1	2415.0	4088.0	1.00			
Female	3191.1	2527.0	4029.9	1.14	1.00	1.30	.053	3207.3	2507.0	4103.3	1.02	0.89	1.17	.761
Age groups														
18–33 years^a^	2682.3	2078.7	3461.0	1.00				2760.1	2111.1	3608.7	1.00			
34–49 years	3008.9	2370.6	3819.0	1.12	0.99	1.27	.072	3099.7	2413.6	3980.9	1.12	0.98	1.28	.083
50–65 years	3309.2	2613.7	4189.8	1.23	1.09	1.40	.001	3739.3	2917.0	4793.3	1.35	1.19	1.54	< .001
Marital status														
Married^a^	2947.5	2330.6	3727.7	1.00				3055.6	2387.9	3909.9	1.00			
Not married	3031.3	2380.1	3860.7	1.03	0.94	1.13	.545	3298.1	2557.3	4253.5	1.08	0.98	1.19	.108
Years of school education														
13 years (A-Level)^a^	3059.7	2401.1	3899.0	1.00				2833.3	2193.2	3660.3	1.00			
10 years	2870.0	2267.9	3632.0	0.94	0.85	1.04	.221	2609.4	2033.0	3349.1	0.92	0.83	1.03	.131
9 years	3123.5	2466.3	3955.8	1.02	0.91	1.15	.731	3620.8	2822.6	4644.8	1.28	1.13	1.44	< .001
No school-leaving certificate	2910.6	2099.8	4034.4	0.95	0.73	1.23	.707	3793.8	2732.5	5267.5	1.34	1.04	1.73	.024
Employment Status														
Employed^a^	2111.4	1923.4	2317.9	1.00				2474.8	2253.9	2717.2	1.00			
Unemployed	4231.7	2693.3	6648.7	2.00	1.27	3.16	.003	4072.2	2522.1	6574.8	1.65	1.01	2.67	.044
Mental distress category (HADS)														
No case^a^	2031.0	1588.2	2597.4	1.00				2259.5	1744.5	2926.6	1.00			
Mild case	2329.7	1810.8	2997.2	1.15	1.03	1.27	.010	2635.1	2024.0	3430.7	1.17	1.05	1.30	.005
Moderate case	3048.4	2389.3	3889.4	1.50	1.36	1.66	< .001	3292.0	2546.0	4256.6	1.46	1.31	1.62	< .001
Severe case	5543.7	4273.8	7167.5	2.73	2.30	3.23	< .001	5181.3	3952.3	6792.3	2.29	1.92	2.74	< .001

*Note. N* = 2.287. *t0* Assessment of predictor variables, *M* Mean non-specific direct costs in Euro per person and year, *CI* 95% confidence interval, *HADS* Hospital Anxiety and Depression Scale. ^a^reference category. Method = log-link function, Tweedie-distribution of residuals

**Table 5:** Results from the generalized linear model to predict the direct specific medical costs (EUR) by self-reported mental distress, controlled for socio-demographic characteristics of the sample

	Specific costs in the first year							Specific costs in the second year						
	M	95% CI		Exp(B)	95% CI		*p*-value	M	95% CI		Exp(B)	95% CI		*p*-value
Constant				93.72	67.94	129.28	< .001				132.74	96.86	181.92	< .001
Gender														
Male^a^	595.4	395.2	897.1	1.00				397.7	259.9	608.6	1.00			
Female	721.4	395.2	897.1	1.21	0.96	1.53	.107	564.9	384.7	608.6	1.42	1.12	1.80	.004
Age groups														
18–33 years^a^	687.6	458.6	1030.8	1.00				464.9	304.7	709.4	1.00			
34–49 years	641.7	439.5	937.0	0.93	0.76	1.15	.515	444.9	298.7	662.6	0.96	0.78	1.17	.673
50–65 years	638.0	443.2	918.6	0.93	0.75	1.14	.484	514.8	349.6	758.1	1.11	0.90	1.36	.330
Marital status														
Married^a^	531.9	367.8	769.2	1.00				414.7	280.7	612.7	1.00			
Not married	807.5	552.8	1179.6	1.52	1.31	1.76	< .001	541.7	363.4	807.5	1.31	1.13	1.52	< .001
Years of school education														
13 years (A-Level)^a^	492.3	334.1	725.4	1.00				474.3	315.0	714.1	1.00			
10 years	648.7	449.0	937.3	1.89	1.04	1.40	.001	487.7	328.0	725.1	1.03	0.87	1.22	.753
9 years	619.4	423.4	906.2	1.26	1.23	1.79	.029	541.4	360.9	812.2	1.14	0.94	1.39	.187
No school-leaving certificate	932.6	573.7	1516.1	1.32	1.10	1.58	.003	403.0	238.8	680.2	0.85	0.55	1.31	.464
Employment Status														
Employed^a^	531.1	458.9	614.7	1.00				409.7	350.6	478.7	1.00			
Unemployed	808.7	402.3	1625.8	1.52	0.76	3.05	.234	548.4	259.3	1159.7	1.34	0.63	2.85	.448
Mental distress category (HADS)														
No case^a^	199.0	134.8	293.9	1.00				213.1	142.2	319.5	1.00			
Mild case	433.6	291.8	644.3	2.18	1.80	2.64	< .001	271.6	179.0	412.2	1.27	1.06	1.53	.010
Moderate case	940.9	647.1	1368.0	4.73	3.97	5.63	< .001	661.1	445.5	980.9	3.10	2.63	3.66	< .001
Severe case	2272.3	1521.0	3394.6	11.42	8.90	14.66	< .001	1318.9	858.7	2026.0	6.19	4.79	7.99	< .001

*Note. N* = 2.287. *t0* assessment of predictor variables, *M* Mean specific direct costs in Euro per person and year, *CI* 95% confidence interval, *HADS* Hospital Anxiety and Depression Scale. ^a^reference category. Method = log-link function, Tweedie-distribution of residuals

#### Age

Compared to 18–33-year-old participants, older participants showed significantly increased non-specific DIW in the first year (*χ*^*2*^[2] = 22.54, *p* < .001, factor 1.2 to 1.5) and the second year (*χ*^*2*^[2] = 31.86, *p* < .001, 1.1 to 1.5). This increase of non-specific DIW was not driven by higher specific DIW (first year: *χ*^*2*^[2] = 2.31, *p* = .315; second year: *χ*^*2*^[2] = 2.23, *p* = .328). Accordingly, older participants showed significantly higher non-specific medical costs in the first year (*χ*^*2*^[2]) = 11.68, *p* = .003) and second year (*χ*^*2*^[2] = 26.83, *p* < .001), but no significant differences in specific medical costs.

#### Marital status

Marital status revealed no significant differences in non-specific and specific DIW in the first or second years. Accordingly, no differences in non-specific medical costs were obtained between married and unmarried participants. The specific medical costs, however, were significantly increased in unmarried participants both in the first year (*χ*^*2*^[1] = 30.56, *p* < .001, factor 1.5) and the second year (*χ*^*2*^[1] = 12.51, *p* < .001, factor 1.3).

#### Education

Lower educated participants showed a significant increase in non-specific DIW. Compared to participants holding an A-Level degree, lower educated participants showed 1.2 to 2.2 as many non-specific DIW in the first (*χ*^*2*^[3] = 33.59, *p* < .001) and in the second year (*χ*^*2*^[3] = 54.60, *p* < .001). The number of specific DIW was also increased for lower educated participants, however, this only yielding significance in the second year. Lower educated participants showed in the first year 1.2 to 2.0 as many specific DIW (*χ*^*2*^[3] = 3.46, *p* = .326) and in the second year 1.4 to 3.3 as many specific DIW (*χ*^*2*^[3] = 11.22, *p* = .011). Regarding the medical costs, no differences were obtained on non-specific costs in the first year, but an increase was shown in non-specific medical costs for lower educated participants in the second year (*χ*^*2*^[3] = 43.12, *p* < .001). An opposite pattern was found concerning specific costs. Here, lower educated participants showed increased costs in the first year (*χ*^*2*^[3] = 14.56, *p* = .002) but not in the second year.

#### Employment status

Employment status revealed no significant differences in non-specific and specific DIW in the first and second years. However, the non-specific costs of unemployed participants were significantly increased. Compared to employed participants, unemployed participants showed 2.0-times as many non-specific medical costs (*χ*^*2*^[1] = 8.99, *p* < .003) in the first year and 1.7-times as many non-specific medical costs (*χ*^*2*^[1] = 4.07, *p* = .044) in the second year. Specific medical costs of unemployed participants were descriptively increased (1.3 to 1.5), but this was not significant.

### Impact of self-reported mental distress

**Fig. 1:**
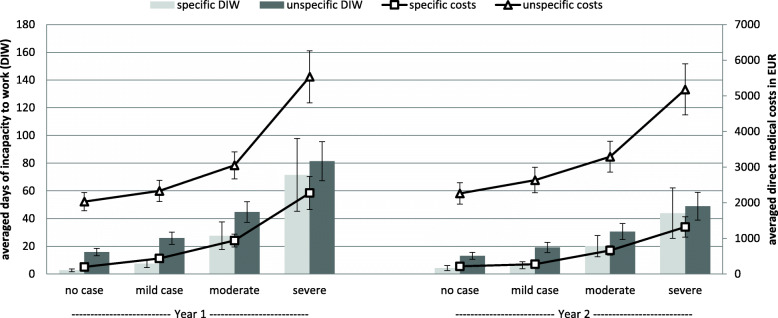
Mean scores of specific and non-specific days of incapacity to work (DIW) on the left y-axis and specific and non-specific medical costs (MC) on the right y-axis in the first and second year after HADS assessment, depending on the HADS severity score. Specific = DIW or MC due to mental illness. Non-specific = DIW or MC due to all diagnoses. Results are controlled for age, gender, marital status, education, and employment status. Error bars represent standard errors

The impact of HADS severity scores on specific and non-specific DIW and medical costs in the first and second years after HADS assessment is depicted in Fig. 1.

#### Days of incapacity to work

Mental distress, as measured with the HADS at t0, showed a significant effect on the number of non-specific DIW in the first year (*χ*^*2*^[3] = 320.78, *p* < .001; see Table 2) and the second year (*χ*^*2*^[3] = 195.17, *p* < .001). The number of non-specific DIW increased continuously with the degree of mental distress. While participants classified as no cases averaged 15.8 non-specific DIW in the first year (13.1 in the second year), mild case participants averaged 1.6 (1.5) times as many non-specific DIW (*M* = 25.8 days in the first year and *M* = 19.1 days in the second year), moderate case participants averaged 2.8 (2.3) times as many non-specific DIW (M = 44.7 days in the first year and *M* = 30.6 days in the second year), and severe case participants averaged 5.1 (3.7) times as many non-specific DIW (*M* = 81.4 days in the first year and 48.9 days in the second year).

This increase of non-specific DIW was mainly driven by an increase of specific DIW in the first year (*χ*^*2*^[3] = 196.98, *p* < .001) and the second year (*χ*^*2*^[3] = 106.21, *p* < .001; see Table 3). While participants, classified as no cases averaged 2.6 specific DIW in the first year (4.3 in the second year), mild case participants averaged 2.8 (1.5) times as many specific DIW (*M* = 7.4 days in the first and *M* = 6.2 days in the second year), moderate case participants averaged 10.5 (4.7) times as many specific DIW (*M* = 27.5 days in the first and *M* = 20.1 days in the second year), and severe case participants averaged 27.3 (10.3) times as many specific DIW (*M* = 71.5 days in the first and *M* = 43.9 days in the second year).

The increases of DIW in the first and second years were obtained on both the anxiety and depression subscales (all *p* values < .001).

#### Medical costs

The increase of DIW depending on mental distress was also reflected in higher non-specific medical costs in the first year (*χ*^*2*^[3] = 164.41, *p* < .001; see Table 4) and the second year (*χ*^*2*^[3] = 110.45, *p* < .001). Again, the number of non-specific medical costs increased continuously according to the degree of mental distress: While participants classified as no cases averaged 2031 EUR non-specific medical costs in the first year (2260 EUR in the second year), mild case participants had 1.2 (1.2) times as many non-specific medical costs (*M* = 2330 EUR in the first year and *M* = 2635 EUR in the second year); moderate case participants had 1.5 (1.5) times as many non-specific medical costs (M = 3048 EUR in the first and M = 3292 EUR in the second year); and severe case participants had 2.7 (1.9) times as many non-specific medical costs (*M* = 5544 EUR in the first and *M* = 5181 EUR in the second year).

The increase of non-specific medical costs was again mainly driven by an increase of specific medical costs in the first year (*χ*^*2*^[3] = 496.56, *p* < 0.001; see Table 5) and the second year (*χ*^*2*^[3] = 309.45, *p* < .001). While participants classified as no cases averaged 199 EUR specific medical costs in the first year (213 EUR in the second year), mild case participants had 2.2 (1.3) times as many specific medical costs (*M* = 434 EUR in the first and *M* = 272 EUR in the second year); moderate case participants had 4.7 (3.1) times as many specific medical costs (*M* = 941 EUR in the first and *M* = 661 EUR in the second year); and severe case participants had 11.4 (6.2) times as many specific medical costs (*M* = 2272 EUR in the first and 1319 EUR in the second year).

The increases in medical costs in the first and second years were obtained on both the anxiety and depression subscales (all *p* values < .001).

## Discussion

This study aimed to examine the impact of self-reported mental distress, assessed by the HADS, on the number of specific and non-specific DIW and medical costs in the 2 years following the testing. To address this aim, we conducted a longitudinal study, in which the HADS scores of 2287 participants were used to predict their specific and non-specific DIW and medical costs in the first and second years after HADS assessment.

Our results revealed that self-reported mental distress (HADS scores) was significantly related to the number of non-specific DIW in the first and second years. Accordingly, the number of non-specific DIW increased continuously based on the level of mental distress. Compared to the reference group classified as no cases, severe cases had 5.1-times as many non-specific DIW in the first year and 3.7-times as many non-specific DIW in the second year. Not surprisingly, the increase of non-specific DIW was mainly driven by a significant increase of specific DIW. Compared to the no cases, severe cases showed 27.3-times as many specific DIW in the first year and 10.3-times as many specific DIW in the second year.

These results demonstrate that mental distress impacts a person’s life for several years by predicting their sickness absence rates even 2 years later. This increased sickness absence rate might be, in turn, related to a generally reduced social and occupational functioning levels and reduced well-being of individuals [55]. Furthermore, mental distress appears to be a central challenge for employers in terms of productivity loss. The financial consequences of specific DIW due to production loss can be calculated by multiplying the specific DIW by average income. Regarding average costs due to production loss in 2014 of 105 EUR per DIW [56], the averaged additional costs for an employee under severe mental distress due to absenteeism alone amount to 7230 EUR in the first years and 4163 EUR in the second year, compared to an employee without mental distress. According to prior empirical findings, the additional costs due to presenteeism can be estimated to be four times higher [53]. Our results revealed that 66% of participants classified as moderate cases and 42% of participants classified as severe cases, did not have any specific DIW in the two-year period that was analyzed. These results indicate that the percentage of people who go to work despite severe mental distress might be considerably high and illustrate the importance and spread of presenteeism. Given this high prevalence of presenteeism and the assumed adverse mental health outcomes, future studies should characterize this sub-sample’s environmental context (e.g. country, cultural norms), work related variables (e.g. job insecurity, strict attendance policies), psychological and personal factors (e.g. consciousness, perfectionism) as well as socio-demographic characteristics (e.g. gender, educational level) to better understand the risk factors of presenteeism [12, 57–59]. By doing so, a distinction should be made between whether work characteristics are perceived as resources and thus contribute to the stabilization of mental health, or as stressors that lead to the maintenance of high mental distress [60].

Both the anxiety and depression subscales of the HADS were predictive for specific and non-specific DIW. This is not surprising since 78% of all specific DIW due to mental illness and burnout in our sample were caused by the diagnostic groups affective disorders (41%, e.g., depression) and neurotic, stress and somatoform disorders (37%, e.g., anxiety disorders). This roughly corresponds to results from other studies in Germany, in which 88.6% of all specific DIW resulted from affective (41.4%) or neurotic, stress, and somatoform disorders (47.2%) [54]. Accordingly, it seems plausible that both the anxiety and depression subscale of the HADS predicted the number of specific DIW in our analyses. However, the impact of both HADS subscales for non-specific DIW is in contrast to the results of Schneider et al. [52], in which only the anxiety symptoms, but not the depressive symptoms, were found to be a significant predictor of the duration of absences due to non-specific DIW.

Beyond non-specific and specific DIW, our results demonstrated that mental distress is also significantly related to individuals’ specific and non-specific medical costs in the first and second year. Specific costs in the first year were 11.4-times higher for severe cases, compared to no cases. Even in the second year, severe cases showed 6.2-times as many specific costs as no cases. This amounts to an additional average specific cost of 2073 EUR per person and year for severe cases in the first year and 1106 EUR per person and year in the second year for the public health care system. The predictive effect of non-specific costs was considerably smaller, but also significant. Compared to no cases, severe cases averaged 2.7-times the costs in the first and 1.9-times the costs in the second year. This amounts to additional average non-specific costs of 3513 EUR per person and year for severe cases in the first year and 2922 EUR per person and year in the second year for the public health care system. The significant relationship between mental distress and non-specific medical costs was mainly driven by specific medical costs. However, these results also imply that subclinical psychiatric symptoms are associated with non-psychiatric medical outcomes. This finding is consistent with other research indicating, for example, an association between psychotic symptoms and diabetes [61], loneliness and venous thromboembolic events [62], or depressive symptoms and blood pressure levels [63].

These results underline the socio-economic burden of mental distress for public health care systems. However, they also show that this burden can be predicted by self-reported mental distress at an early stage. This result is consistent with previous studies, which have identified subclinical symptoms in the general population as predictive for later mental disorders [21–29]. There is rising evidence for the importance of subclinical symptoms to recognize possible mental burden at an early stage and opening the possibility of prevention.

Although our data represent costs from a German population, these results can be seen as an indicator for other industrialized countries, since both the prevalence of mental disorders (Germany 18%, EU 17.3%) and the percentage of direct and indirect medical costs due to mental illness in Germany (Germany 4.8%, EU 4.0%) are comparable to other EU countries [9].

Most demographic characteristics of our sample showed no consistent effects across the different dependent variables. However, these have been included mainly as control variables to control possible confounding variables. Future studies should specifically focus on these variables to draw reliable conclusions about socio-demographic variables’ influence on absence days and medical costs. Only the participants’ age showed a consistent pattern with increased non-specific DIW and non-specific medical costs for both years, but no differences in specific DIW and specific medical costs. Lower education in our sample was significantly related to non-specific DIW. However, on specific DIW, the increase by lower education yielded significance only in the second year. These results are in line with prior studies showing that mental distress (anxiety symptoms), higher age, and lower education emerged as significant predictors of non-specific DIW [52]. Given these findings, it seems likely that lower educational status and higher age can be considered a risk factor for non-specific DIW. However, their effect on specific DIW or medical costs remains uncertain. Future studies should include large and representative samples to investigate the differential effects of age and education on specific and non-specific DIW and specific and non-specific medical costs.

Contrary to prior studies, in which female gender was found to be a significant predictor of specific DIW [53, 54], our analyses showed no differences of specific DIW between male and female participants. However, a closer look at the descriptive factors shows that the factors from our study (1.57) are comparable to those from previous studies (1.6) [53, 54]. Therefore, the non-significant differences in DIW depending on the sample characteristics in our study could result from a too-small sample size in the different subgroups, thus limiting the power for individual comparisons. With 89%, the proportion of female participants was considerably high. Interestingly, female participants showed higher specific medical costs in both years. This finding is in line with prior research, indicating a higher prevalence of anxiety and affective mental disorders in female populations [9, 64].

### Strengths, limitations, and recommendations for future research

Our study’s major strengths relate to its longitudinal research design and the analysis of real DIW and medical cost data from a health insurance company in conjunction with psychometrically assessed mental distress from individuals. By including DIW and medical costs in the first and second year, we were able to show that self-reported mental distress was predictive for DIW and medical costs regardless of the DIW and medical costs occurring immediately after the HADS assessment, and this enabled us to show the long-term consequences of severe mental distress. By including specific and non-specific DIW and specific and non-specific costs as dependent variables, we were able to show the importance of mental health for general, occupational functioning and point to the consequences of mental distress for companies and the public health care systems. Furthermore, the available cut-off scores of the HADS to distinguish between no, mild, moderate, and severe cases allowed us to demonstrate clear, practical implications for the consequences of severe mental distress in applied settings. However, this study has some limitations, which should be considered when interpreting the results.

Firstly, we only investigated the main effects of the sample characteristics and mental distress. However, more complex interaction effects between the independent variables are conceivable and should be investigated in future studies using larger sample sizes. Secondly, although our total sample was reasonably large, it is not a representative sample of the German population. Accordingly, we have included sociodemographic variables including sex, age groups, employment status, education and marital status in the models. However, some socio-demographic subgroups might be too small, resulting in limited power to retrieve reliable conclusions about the effect of different sample characteristics on DIW and medical costs, respectively (e.g., *n* = 11 participants in the unemployment group). Third, in addition to the sample characteristics analyzed in this study, other variables might impact the relationship between mental distress and DIW, such as the quality of health management in organizations [65], subjectively perceived workplace characteristics (e.g., social support, leadership quality [66, 67]), or inter-individual differences in psychological conditions, such as self-efficacy or work attitude [68]. In addition, variables should be investigated, influencing the relationship between mental distress and medical costs, such as access to psychotherapy or stigmatization. Finally, we analyzed DIW and medical costs independently of each other. Given possible confounding factors between those dependent variables, a multivariate analysis would have been appropriate to control for these dependencies. However, due to the Tweedie distributions of both the cost and DIW data, which represents a serious deviation from the normal distribution, the prerequisite for a multivariate analysis was not given. The use of general linear models allowed us to model Tweedie distributions. Future studies should investigate how medical costs and DIW are related to each other over time (e.g., whether increased specific medical costs help reduce DIW). Future studies should also systematically investigate how prevention programs for distressed individuals and evidence-based treatments for individuals with mental disorders contribute to saving money by restoring occupational and social functioning.

### Implications for practice

This study shows the extent to which self-reported mental distress is related to the subsequent inability to work and to medical costs. On an individual level, our results indicate that mental distress affects a person’s life after a span of 2 years by reducing occupational and social functioning. On a societal level, our results demonstrate the high socio-economic costs of mental distress through productivity losses due to reduced functional levels. The results, therefore, suggest that joint efforts should be made to effectively reduce mental distress [69]. Individuals with mild and moderate mental distress who do not yet suffer from a manifested mental illness should be given access to preventive services [70]. Preventive structures should be established within peoples’ everyday lives (e.g., at the workplace) to enable low-threshold access [15, 16]. Workplace health promotion programs play a special role here because occupational risk factors, such as emotional load or work-related stress, can contribute critically to increased mental distress, which often manifests itself in anxiety and depression symptoms as well as burnout conditions [71, 72]. This also refers to the importance of occupational health surveillance for the monitoring and early prevention of mental distress, since not recognizing mental distress, ignoring it, or not taking effective countermeasures might exacerbate the problem and result in significant negative financial impact [19, 20, 34, 73]. A preventive commitment from employers to the workforce’s mental health should ultimately lead to a better working atmosphere, a better quality of life for employees, and an increase in productivity [11].

Individuals with severe mental distress or those with manifested mental disorders should be given rapid access to specialized help in the form of evidence-based psychotherapeutic or psychiatric treatments [31]. Prior studies from the UK have shown that increasing access to psychological therapies would largely pay for itself by reducing other depression and anxiety-related public costs (e.g., medical costs and productivity loss) and increasing revenues (e.g., paying taxes [32]). Rapid access to mental health services should be enabled, since the time spent waiting to start psychological treatments was negatively associated with treatment outcome [31].

## Conclusion

In summary, our study demonstrates the extent to which mental distress is associated with reduced occupational and social functioning. Accordingly, mental distress significantly impacts the number of DIW and medical costs for a span of 2 years following the initial HADS assessment. These results indicate that improving preventive structures for at-risk populations and increasing access to specialized treatments for individuals with mental disorders might reduce individual suffering as well as public costs.

## Data Availability

The datasets generated and/or analyzed during the current study are not publicly available due to the data protection policy from the cooperating insurance fund but are available from the corresponding author on reasonable request (in anonymized form).

## Abbreviations

DIW: Days of Incapacity to Work
HADS: Hospital Anxiety and Depression Scale
EUR: Euro
DALY: Disability Adjusted Life Years
EU: European Union
US: United States
USD: US Dollar
IAPT: English Improving Access to Psychological Therapies
UK: United Kingdom
DRKS: Deutsches Register Klinischer Studien (German Register of Clinical Studies)
ICD: International Statistical Classification of Diseases and Related Health Problems
SPSS: Statistical Package for Social Science
